# Supplementary material for: In situ synthesis of supported metal nanocatalysts through heterogeneous doping
Source: Nat Commun. 2018 Nov 16;9:4829. doi: 10.1038/s41467-018-07050-y (PMC6240097; doi:10.1038/s41467-018-07050-y)
Supplement: Supplementary file 2 — Supplementary Information [file 41467_2018_7050_MOESM2_ESM.pdf]

Supplementary Information

In situ synthesis of supported metal nanocatalysts through heterogeneous doping

No Woo Kwak,[1] Seung Jin Jeong,[1] Han Gil Seo,[1] Siwon Lee,[1] YeonJu Kim,[1] Jun Kyu Kim,[1] Pilgyu Byeon,[1] Sung-Yoon Chung[1] & WooChul Jung*[1]

[1] Dep. of Materials Science and Engineering, Korea Advanced Institute of Science and Technology, 291 Daehak-ro, Yuseong-gu Daejeon, 34141, Republic of Korea.

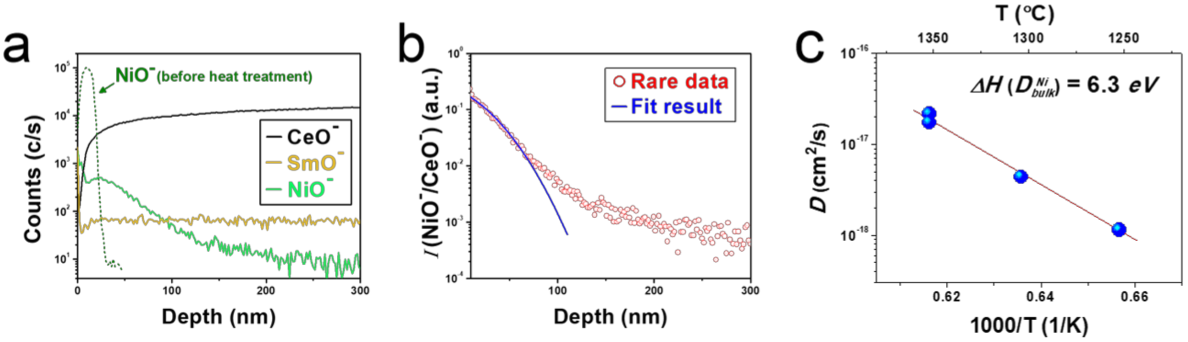

Supplementary Figure 1. (a) Time-of-flight secondary ion mass spectroscopy (ToF-SIMS) depth profiles of CeO^-, SmO^-, and NiO^- into Sm 0.5 at % doped CeO_2 bulk pellet before/after heat treatment at 1350°C during 50 hours in ambient air and (b) ((NiO^-)/(CeO^-)) and fit result (solid line) as a function of sputtering depth. (c) The obtained values and activation energy of diffusion coefficient of Ni inside ceria bulk lattice in air.

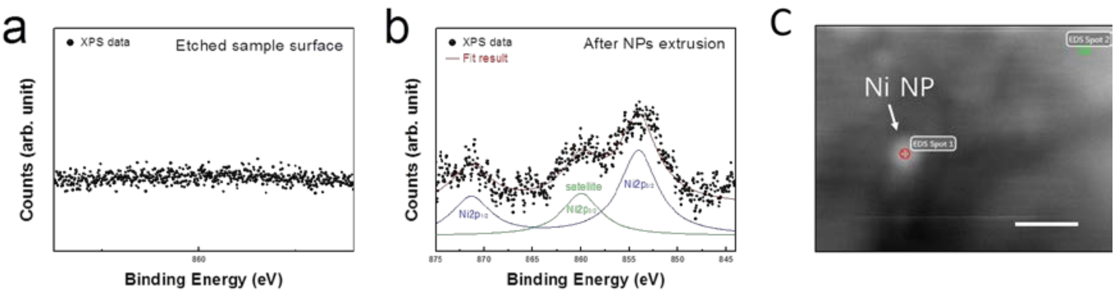

Supplementary Figure 2. X-ray photoelectron spectroscopy (XPS) spectra from Sm-doped CeO_2 (SDC) surfaces after (a) etching the sample surface and (b) extrusion of Ni nanoparticles. (c) Field-emission scanning electron microscope (FE-SEM) image of Ni nanoparticle on ceria surface (scale bar = 50 nm).

Supplementary Table 1. Energy dispersive spectroscopy (EDS) results of spots 1 and 2 from Supplementary Figure 2c.

	Spot 1 (at %)	Spot 2 (at %)
Ce	75	84
Sm	13	15
Ni	12	1

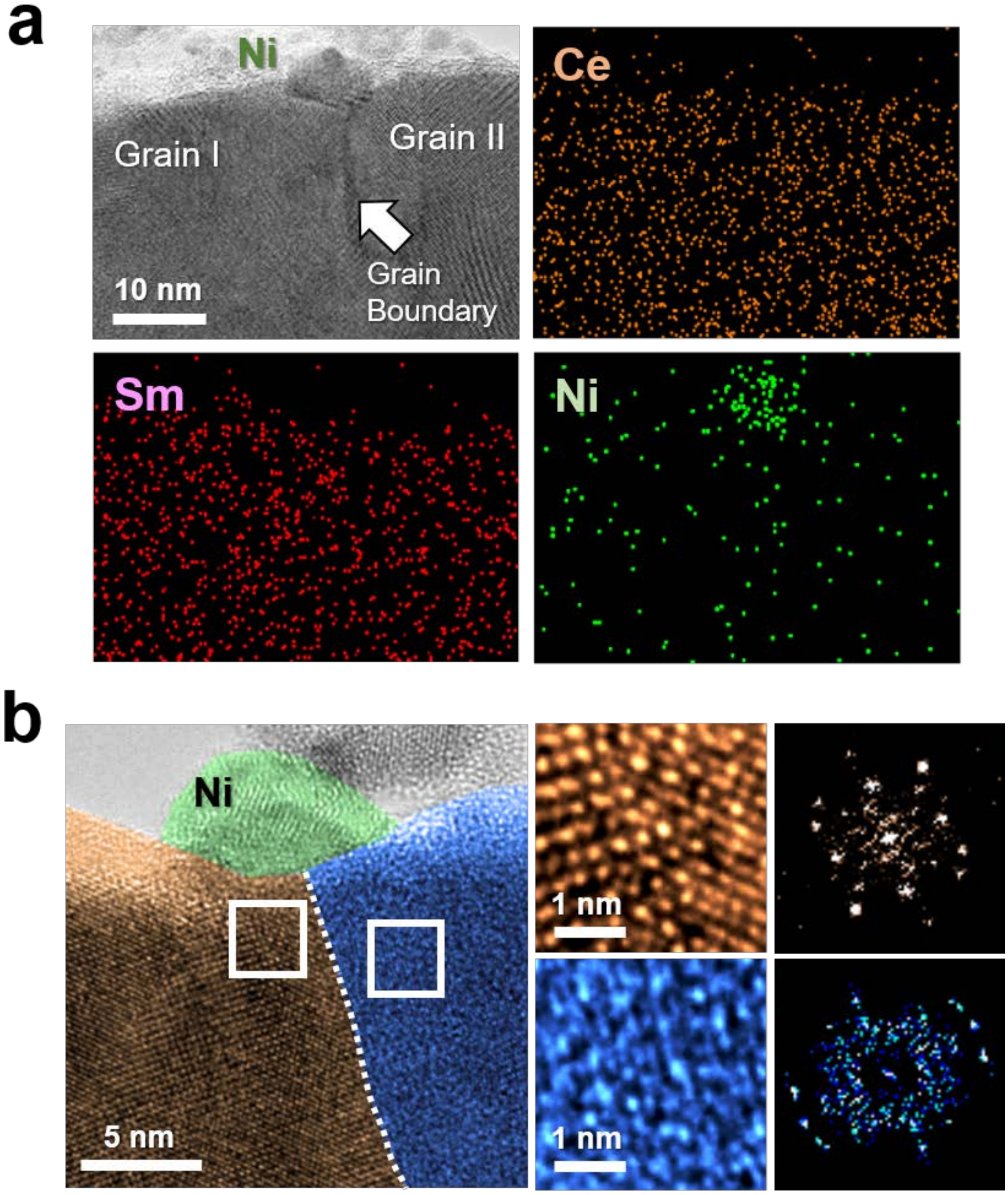

Supplementary Figure 3. (a) Cross-sectional scanning transmission electron microscope (STEM) image and energy dispersive X-ray spectroscopy (EDS) mapping results of SDC film with a Ni particle. (b) The enlarged high-resolution electron microscopy (HREM) images and corresponding Fast Fourier transform (FFT) patterns of adjacent grains.

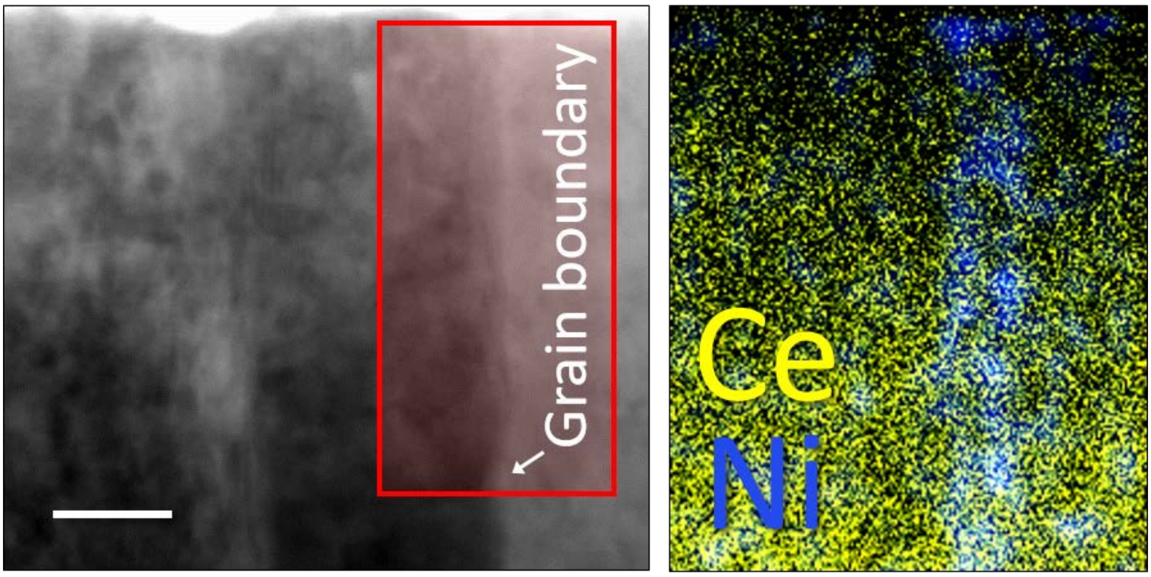

Supplementary Figure 4. Cross-sectional scanning transmission electron microscope (STEM) image of Samarium doped CeO_2 (SDC) film and Energy Dispersive X-ray Spectroscopy (EDS) mapping results of the highlighted area marked in red (scale bar = 20 nm).

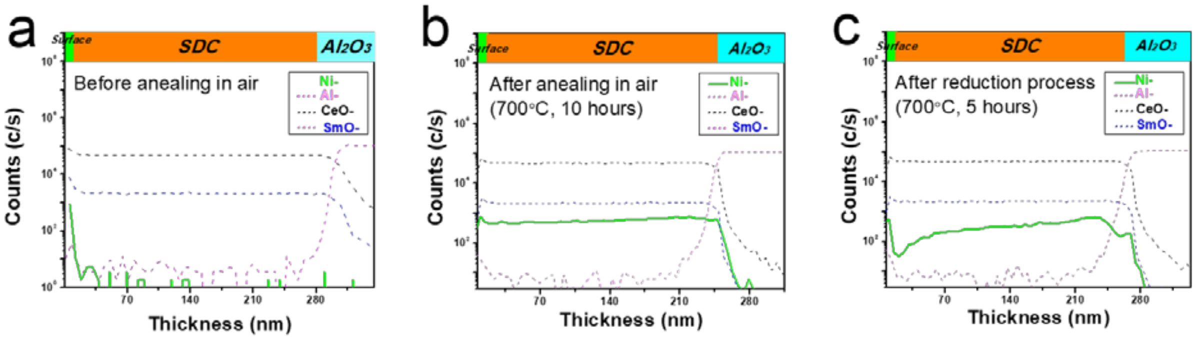

Supplementary Figure 5. Depth profiles of Ni^-, Al^-, SmO^-, and CeO^- from secondary ion mass spectroscopy (SIMS) measurements (a) before and (b) after annealing in air, and (c) after reduction process (the intensity of Ni^- increases near top surface because Ni NPs are existed on film surface).

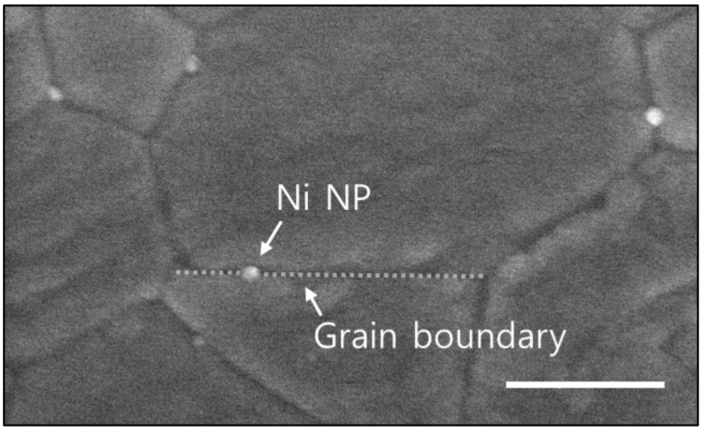

Supplementary Figure 6. Field-emission scanning electron microscope (FE-SEM) image of ceria bulk pellet surface after extrusion of Ni nanoparticles at 700°C (scale bar = 500 nm).

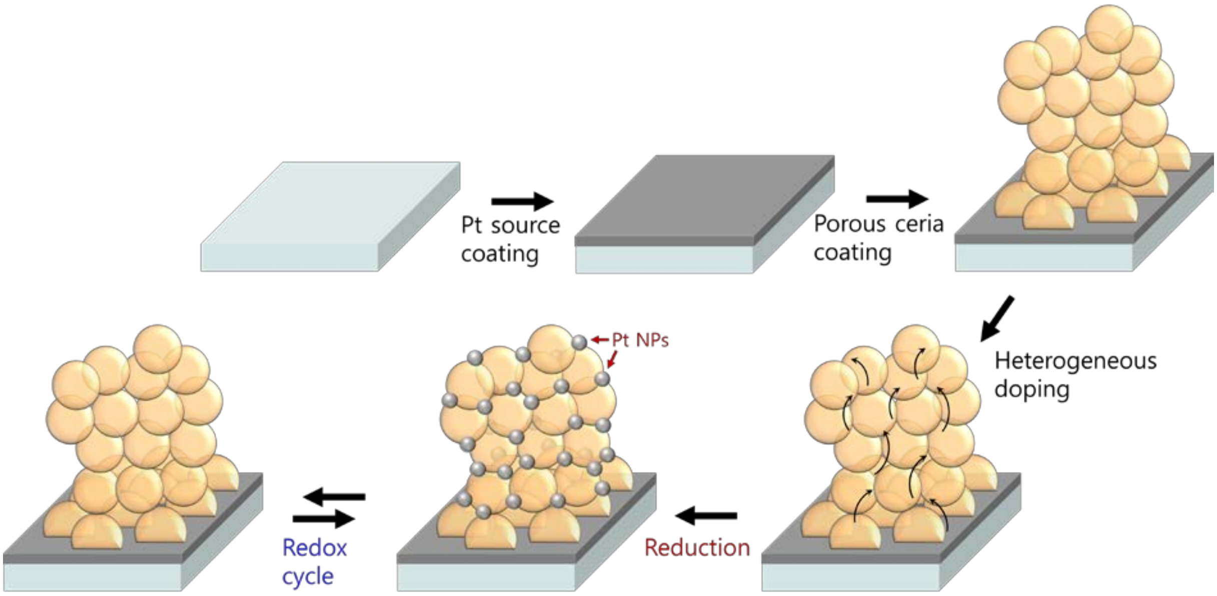

Supplementary Figure 7. Schematic diagram of the overall synthesis procedures to form Pt nanoparticles on porous ceria surfaces. Pt atoms move back and forth between the interior (as cations) and the exterior (as clusters) of the host ceria as the reductive and oxidative atmospheres repeat.

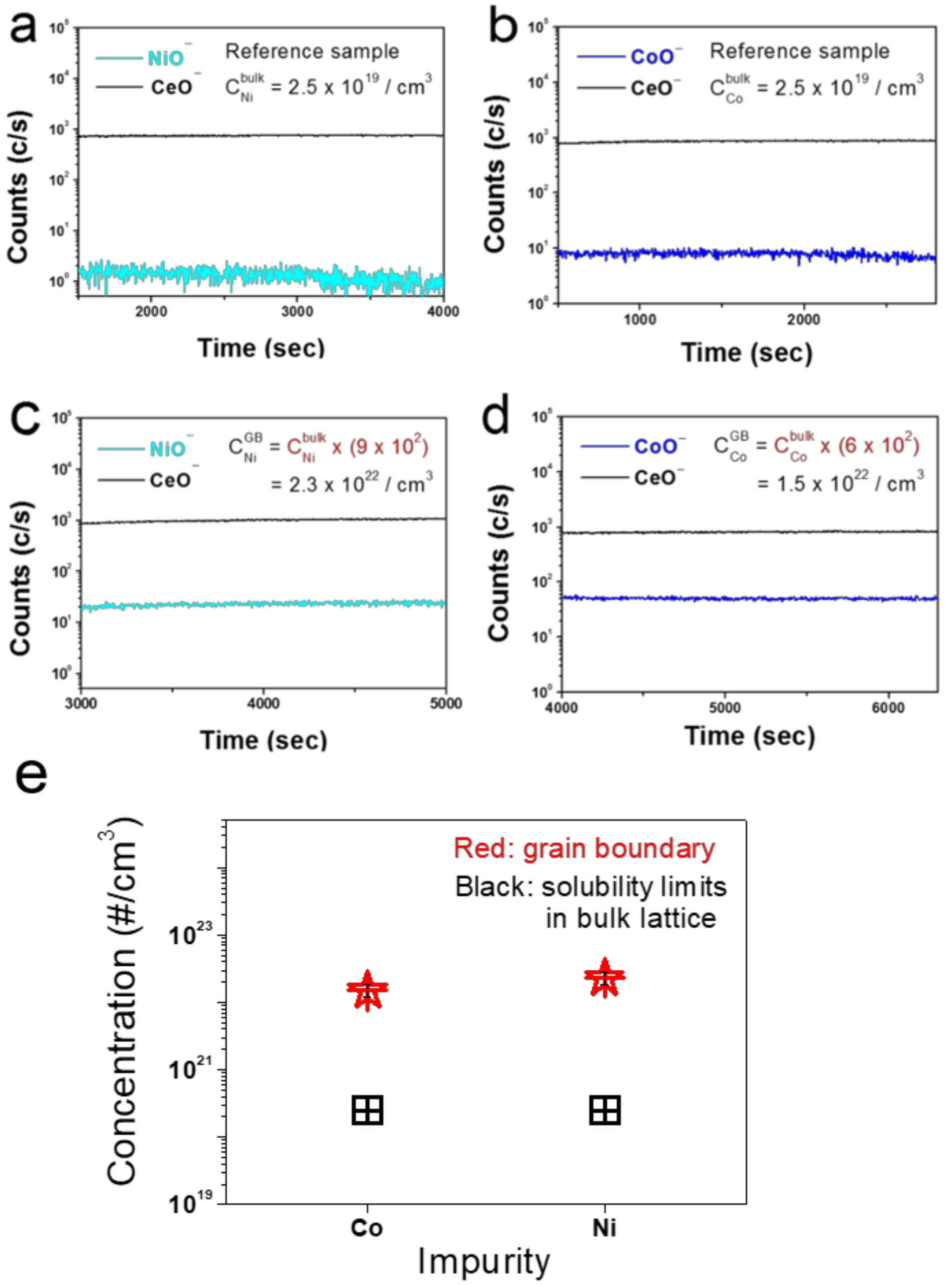

Supplementary Figure 8. Time-of-flight secondary ion mass spectroscopy (ToF-SIMS) depth profiles of secondary ions from (a) Ni-doped (0.1 at %) and (b) Co-doped (0.1 at %) reference samples, and heterogeneously (c) Ni-doped and (d) Co-doped ceria thin films. (e) Comparison of the solubility limits of transition metals (Co and Ni) in the bulk and grain boundaries of CeO_2, measured at 700°C

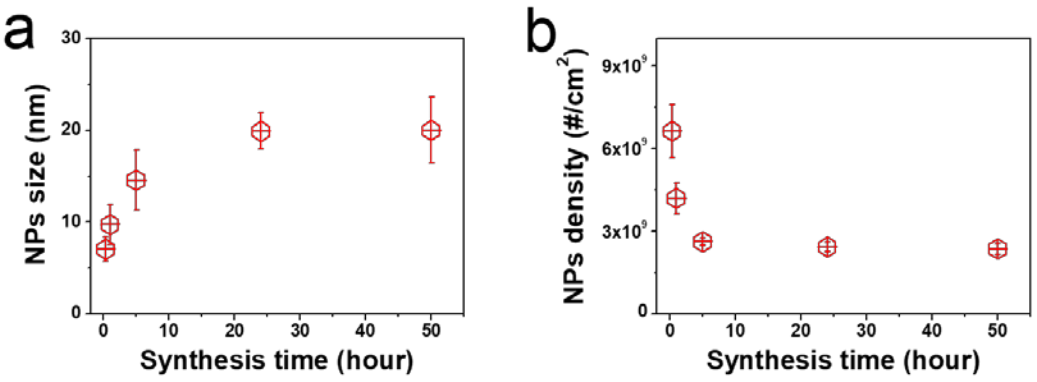

Supplementary Figure 9. The change of (a) size and (b) number density of Ni NPs as a function of synthesis time at 700°C.

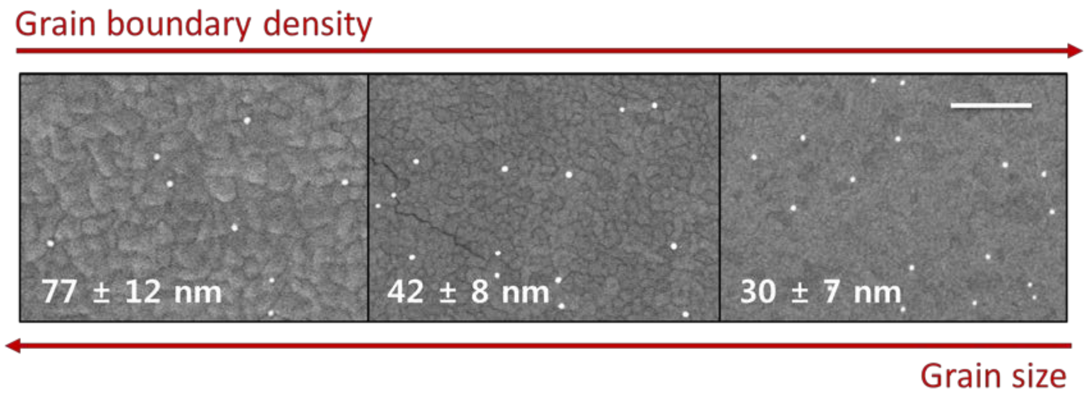

Supplementary Figure 10. Size-distribution change of Ni nanoparticles with grain boundary density difference of SDC films. Numbers inside images indicate the grain size of the ceria films (scale bar = 300 nm).

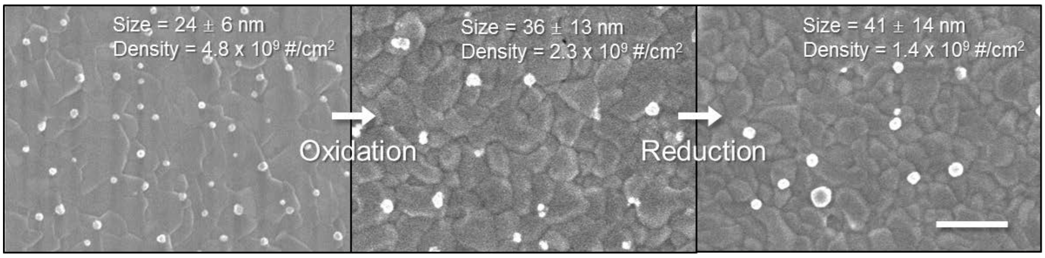

Supplementary Figure 11. Field-emission scanning electron microscope (FE-SEM) images showing an unstable tendency of Ni nanoparticles produced by the sputtering method according to redox cycles at 700°C (scale bar = 300 nm).

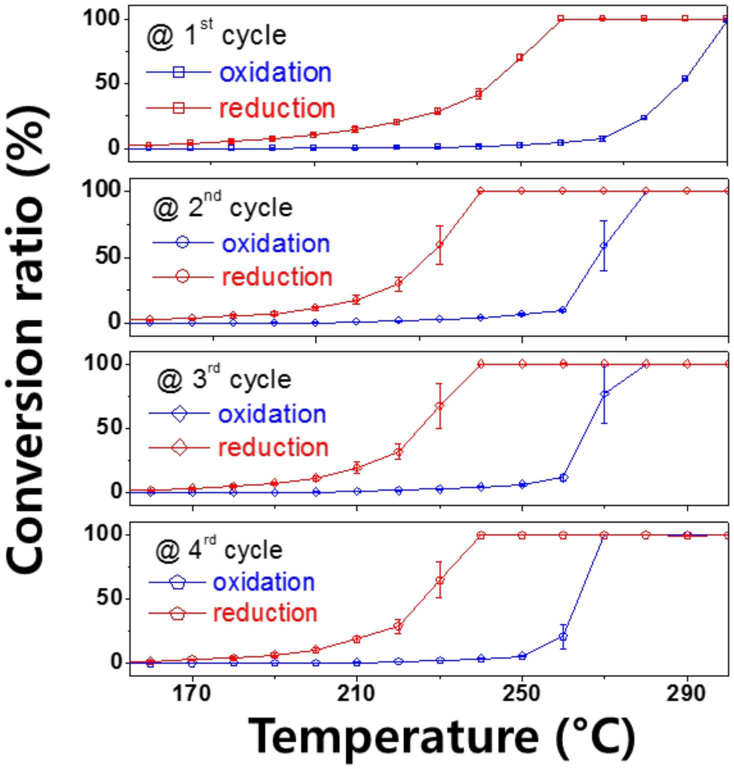

Supplementary Figure 12. Catalytic activity curves of CO_2 conversion with respect to the temperature for porous ceria according to redox cycles.

Supplementary Notes

Supplementary Note 1. Derivation Process of Diffusion Solution

If the region $0 < x < l$ is initially at a uniform concentration, C_0, and the surfaces are kept at a constant concentration, C_{surf}, the solution of concentration of out-diffusing species at time t and locus x, $C_{(x,t)}$, becomes[1]

$$\frac{C_{(x,t)} - C_0}{C_{surf} - C_0} = \left[1 - \frac{4}{\pi}\sum_{n=0}^{\infty}\frac{(-1)^n}{2n+1}\exp\left\{-D(2n+1)^2\pi^2\frac{t}{4l^2}\right\}\cos\frac{(2n+1)\pi x}{2l}\right] \tag{1}$$

where l is the sample thickness, D the diffusion coefficient, and t the time.

Also, by the mass conversion principle, the total volume of NPs on the surface during ex-solution, $V_{(t)}$, becomes the sum of volume of out-diffused species from host oxide, which is expressed as follows,

$$V_{(t)} = \frac{S}{\rho}\int_0^l \left(C_0 - C_{(x,t)}\right)dx \tag{2}$$

where S is the surface area of the sample and ρ the density of NPs.

By combining Supplementary Equations 1 and 2, we obtain that

$$\begin{aligned} V_{(t)} &= \frac{S}{\rho}\left(C_0 - C_{surf}\right)\int_0^l \left[1 - \frac{4}{\pi}\sum_{n=0}^{\infty}\frac{(-1)^n}{2n+1}\exp\left\{-D(2n+1)^2\pi^2\frac{t}{4l^2}\right\}\cos\frac{(2n+1)\pi x}{2l}\right]dx \\ &= \frac{S}{\rho}\left(C_0 - C_{surf}\right)\left[l - \frac{4}{\pi}\sum_{n=0}^{\infty}\frac{(-1)^n}{2n+1}\exp\left\{-D(2n+1)^2\pi^2\frac{t}{4l^2}\right\}\int_0^l \cos\frac{(2n+1)\pi x}{2l}dx\right] \\ &= \frac{S}{\rho}\left(C_0 - C_{surf}\right)\left[l - \frac{8l}{\pi^2}\sum_{n=0}^{\infty}\frac{(-1)^n}{(2n+1)^2}\exp\left\{-D(2n+1)^2\pi^2\frac{t}{4l^2}\right\}\sin\frac{(2n+1)\pi l}{2l}\right] \\ &= \frac{lS}{\rho}\left(C_0 - C_{surf}\right)\left[1 - \frac{8}{\pi^2}\sum_{n=0}^{\infty}\frac{1}{(2n+1)^2}\exp\left\{-D(2n+1)^2\pi^2\frac{t}{4l^2}\right\}\right] \end{aligned} \tag{3}$$

The fitting result (dot line) using Supplementary Equation 3 with the total volume change of extruded Ni NPs as a function of synthesis time is shown in Fig. 4b.

Supplementary Note 2. Interpretation of Impedance Spectra

Nyquist plots of AC impedance results for H_2 electro-oxidation in this study always consists of a nearly one ideal semicircle, displaced from the origin by a high-frequency offset resistance ($R_{off\text{-}set}$). The high-frequency offset is attributed to a combination of the ionic resistance of the YSZ and the sheet resistance of the current collector. The low-frequency arc reflects the

characteristics of the electrochemical reaction and is modeled using a *RQ* subcircuit as reported in previous work.[2]

Supplementary References

1. Crank, J. The Mathematics of diffusion, Oxford, 1975.

2. Choi, Y. et al. Electrochemically modified, robust solid oxide fuel cell anode for direct-hydrocarbon utilization. *Nano Energy*. 23, 161-171 (2016).
